# GEN2VCF: a converter for human genome imputation output format to VCF format

**DOI:** 10.1007/s13258-020-00982-0

**Published:** 2020-08-16

**Authors:** Dong Mun Shin, Mi Yeong Hwang, Bong-Jo Kim, Keun Ho Ryu, Young Jin Kim

**Affiliations:** 1 Division of Genome Research, Center for Genome Science, National Institute of Health, Osong Health Technology Administration Complex, 187, Osongsaengmyeong 2-ro, Osong-eup, Heungdeok-gu, Cheongju-si, Chungcheongbuk-do 28159 Republic of Korea; 2 Data Science Laboratory, Faculty of Information Technology, Ton Duc Thang University, Ho Chi Minh City, 700000 Vietnam; 3 Database and Bioinformatics Laboratory, Department of Computer Science, College of Electrical and Computer Engineering, Chungbuk National University, 28644 Cheongju, Republic of Korea

**Keywords:** Human genome, Imputation, SNP, Converter, Parsing

## Abstract

**Background:**

For a genome-wide association study in humans, genotype imputation is an essential analysis tool for improving association mapping power. When IMPUTE software is used for imputation analysis, an imputation output (GEN format) should be converted to variant call format (VCF) with imputed genotype dosage for association analysis. However, the conversion requires multiple software packages in a pipeline with a large amount of processing time.

**Objective:**

We developed GEN2VCF, a fast and convenient GEN format to VCF conversion tool with dosage support.

**Methods:**

The performance of GEN2VCF was compared to BCFtools, QCTOOL, and Oncofunco. The test data set was a 1 Mb GEN-formatted file of 5000 samples. To determine the performance of various sample sizes, tests were performed from 1000 to 5000 samples with a step size of 1000. Runtime and memory usage were used as performance measures.

**Results:**

GEN2VCF showed drastically increased performances with respect to runtime and memory usage. Runtime and memory usage of GEN2VCF was at least 1.4- and 7.4-fold lower compared to other methods, respectively.

**Conclusions:**

GEN2VCF provides users with efficient conversion from GEN format to VCF with the best-guessed genotype, genotype posterior probabilities, and genotype dosage, as well as great flexibility in implementation with other software packages in a pipeline.

## Introduction

A genome-wide association study (GWAS) is a well-known approach to identify genetic variations associated with complex traits (Visscher et al. 2012). The GWAS Catalog is a free online database that collects GWAS results. As of November 2019, the catalog contains 161,525 variant-trait associations from 4298 publications (https://www.ebi.ac.uk/gwas/) (Buniello et al. 2019). In a GWAS, genotype imputation has been regarded as an essential analysis tool to improve the power of association mapping by estimating tens of millions of variants that are not directly genotyped using a single nucleotide polymorphism (SNP) microarray. Genotype imputation infers missing or untyped SNPs in a study dataset from a reference panel, such as the 1000 Genomes project and Haplotype Reference Consortium (Auton et al. 2015; Huang et al. 2009; McCarthy et al. 2016). Various imputation tools have been introduced such as IMPUTE2 (Howie et al. 2009), BEAGLE (Browning and Browning 2016), Mach (Li et al. 2010), and Minimac (Howie et al. 2012).

By default, imputation estimates posterior probabilities of three genotypes AA, AB, and BB. These posterior probabilities are often used in a form of three different types in association testing: the best-guessed genotype (GT) with maximum posterior probability; genotype probabilities (GPs); and genotype dosage (DS), which is the posterior mean of three posterior probabilities. Among them, DS is widely used in testing associations for imputed genotypes. The association test using DS showed enhanced statistical power (Liu et al. 2013).

However, there are challenges in using imputed dosages in association tests. Dedicated software packages, such as SNPTEST (see URLs) and mach2qtl (see URLs), using imputed dosages in association testing does not support various statistical methods and gene-based tests supported by recent association software packages, such as EPACTS (see URLs) and RAREMETAL (Feng et al. 2014). EPACTS and RAREMETAL are used to perform various statistical analyses and gene-based association tests using variant call format (VCF), which contains formatted imputed genotypes. Although the recently developed Minimac 3 outputs imputation data in a VCF file, IMPUTE only outputs GEN files, a non-VCF file (Howie et al. 2012). Even though IMPUTE does not support VCF, IMPUTE has been widely used in many GWASs due to its high imputation accuracy comparable to Minimac (Das et al. 2016). To handle imputed data from IMPUTE, an additional conversion process is required for subsequent association analyses.

Existing tools that support a VCF conversion process, such as BCFtools (see URLs) and QCTOOL (see URLs), convert IMPUTE GEN files to VCF without dosage information. Thus, additional data processing using VCF parsers, such as PySAM (see URLs), is required to obtain dosage information, and the output can be merged with VCF data from BCFtools and QCTOOL. Oncofunco is an R package (see URLs) that converts posterior probabilities in an IMPUTE2 gen file to dosage and then outputs to a VCF file. The VCF file contains only dosage information; therefore, other information is added using the VCF parser. These multiple conversion steps may take a lot of time for reading, modifying, and writing data. Currently, as far as we know, Hail (see URLs) is the only software package that can be used for converting GEN files to VCF files. Hail uses Spark to read and write large data sets (Ganna et al. 2016; Khera et al. 2018). However, the implementation of a Spark-based system environment requires experts in related fields and a supercomputing resource for handling a large-scale dataset. Therefore, a fast and convenient GEN format to VCF conversion tool with DS support is warranted.

In this paper, we present a new tool GEN2VCF, which converts the IMPUTE output in GEN format to VCF. GEN2VCF provides DS as well as GT and GP. GEN2VCF is a C-based software that converts GEN files faster than the existing pipelines and is efficient in handling large amounts of data with low memory usage. GEN2VCF also has options for standard input and output of processing data. This feature is particularly useful in implementing GEN2VCF with various different software packages by piping and redirection. We compared the performance of GEN2VCF with three possible pipelines by using combinations of three converting tools (BCFtools, QCTOOL, and Oncofunco) and a VCF parser (PySAM). A subset of chromosome 1 of the imputed data of 5000 samples was used as input data. To measure the performance, runtime and memory usage were used as measures.

## Materials and methods

### Implementation of GEN2VCF

GEN2VCF was implemented in the C programming language on Linux-based operating systems, which allows for large amounts of imputed data to be handled quickly. Memory usage is also relatively low compared to other programming languages (Fourment and Gillings 2008). All GEN2VCF commands are run in a Linux terminal. Given two alleles of A, B, there are three possible genotypes of a SNP: AA, AB, and BB. The A allele was regarded as the reference allele, and B allele as a coded allele (alternative allele). From the imputation output, the probability of each genotype is given by P(AA), P(AB), and P(BB). An imputed genotype dosage was estimated as 0 · P(AA) + 1 · P(AB) + 2 · (BB) (Hoffmann and Witte 2015). The dosage has a value between 0 and 2.

## Comparison with other existing software packages

**Fig. 1:**
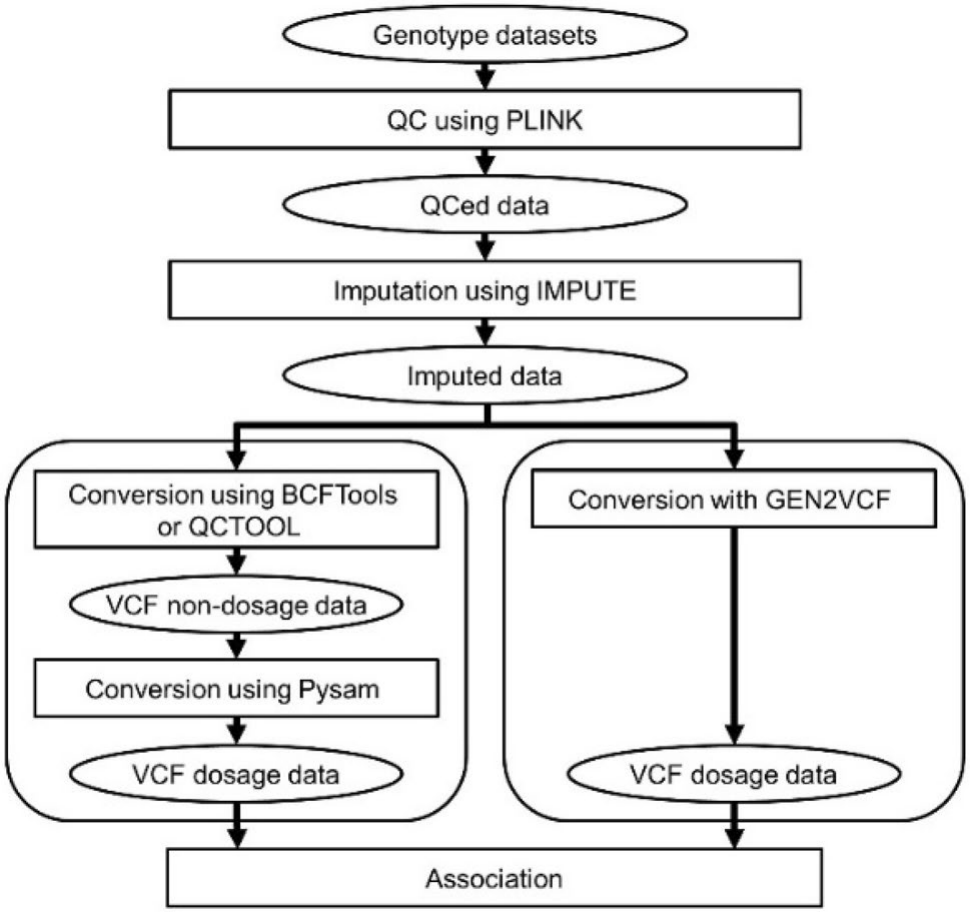
Conversion processes of GEN2VCF and existing software packages

For the comparison analysis, we converted a GEN-formatted file, which is an output from IMPUTE software, to a VCF file with GT, GP, and DS. In the conversion from GEN format to VCF, the processes of GEN2VCF and existing software packages (BCFtools, QCTOOL, and Oncofunco) were displayed in Fig. 1. Briefly, there are three main steps during conversion processes: (1) the GEN file generated by IMPUTE is read, (2) dosages are calculated using genotype probabilities in the GEN file, and (3) an indexed compressed (bgzip) VCF file with GT, GP, and DS is generated. The basic characteristics of GEN2VCF and existing software packages are summarized in Table 1. Since the existing software alone do not have an option for handling dosage values ​​for the conversion, an imputed genotype dosage was calculated using the VCF parser PySAM. On the other hand, GEN2VCF provides the conversion in a single process, thereby enabling more efficient analysis.

**Table 1:** Basic characteristics of methods used in this study

Tool	Input	Output	VCF FORMAT Field	Ver.	Ref.	URL
BCFtools	GEN, BCF, VCF, HAPS	BCF, VCF	GT, PL, GL, GQ, GP	1.6	PMID: 21,903,627	http://samtools.github.io/bcftools/bcftools.html
QCTOOL	GEN, BGEN, VCF	GEN, VCF	GT, GP	1.4	doi: 10.1101/308296	http://www.well.ox.ac.uk/~gav/qctool/#overview
GEN2VCF	GEN, BGEN	VCF	GT, GP, DS	1.0	–	https://bitbucket.org/4shin/division-of-genome-research/src/master
Oncofunco	GEN	VCF	DS	–	–	https://github.com/oncogenetics/oncofunco

## Performance test

For the experiment, we randomly sampled imputed data from a 1 Mb region on chromosome 1 from 5000 samples that was previously genotyped with the Korea Biobank Array (Moon et al. 2019). The 1 Mb genotype data were pre-phased using Eagle v2.3 (Loh et al. 2016) and imputed using Impute v4 (Bycroft et al. 2018) using the 1000 Genomes project phase 3 data as a reference panel (Auton et al. 2015). The imputed dataset consists of 13,891 variants. All experiments were performed on a computer with an Intel Xeon processors 3.47 GHz (12 cores), 66 GB of memory, and the Linux-based operating system Ubuntu 14.04.6. To measure the performances of GEN2VCF and other software packages, we used total runtime and maximum memory usage as performance measures. All tools were used with their default options in a single process.

## Results

We performed a comparison analysis between GEN2VCF and possible three existing pipelines by using combinations of three converting tools (BCFtools, QCTOOL, and Oncofunco) and a VCF parser (PySAM). We converted a GEN-formatted file, which was an output from the IMPUTE software, to a VCF file with GT, GP, and DS. To determine the performance for various sample sizes, tests were performed from 1000 to 5000 samples with a step size of 1000. To determine the performance, total runtime and memory usage was used for each approach.

The basic characteristics of the four methods used in this study are summarized in Table 1. BCFtools and QCTOOL only support the GT and GP of each genotype. Oncofunco outputs a VCF file with DS except GT and GP. Therefore, the VCF parser PySAM was used to combine VCF files with partial information to generate a VCF file with GT, GP, and DS.

**Fig. 2:**
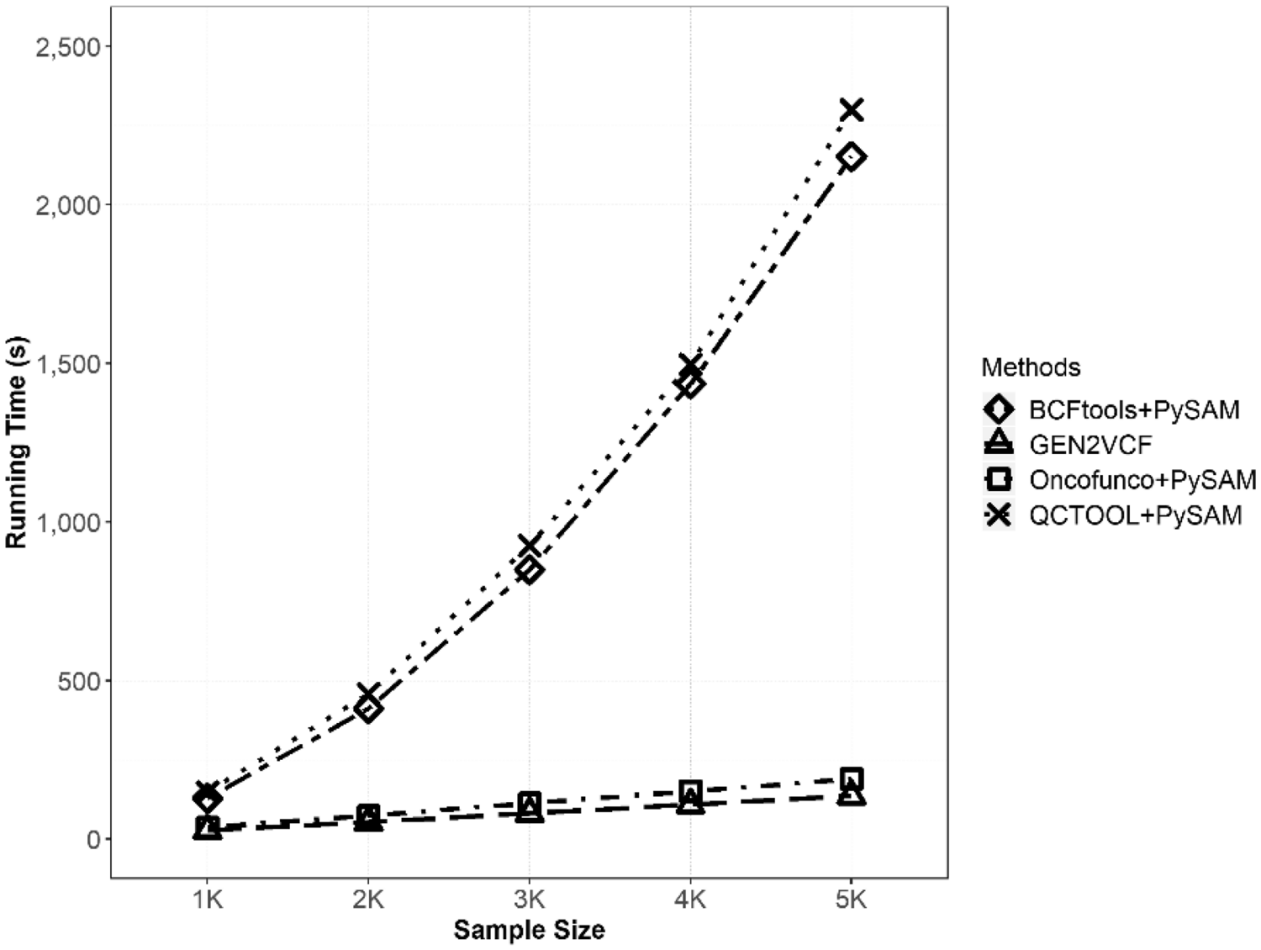
Runtime comparison among the four methods

Figure 2 shows the total runtime of each method. As shown in the figure, GEN2VCF was the fastest among the four methods. The second fastest pipeline was Oncofunco and BCFtools used with PySAM. The runtime for generating a VCF file using QCTOOL and PySAM was the lowest of the four. However, GEN2VCF showed a 1.4–17-fold decrease in conversion time compared to the other pipelines.

**Fig. 3:**
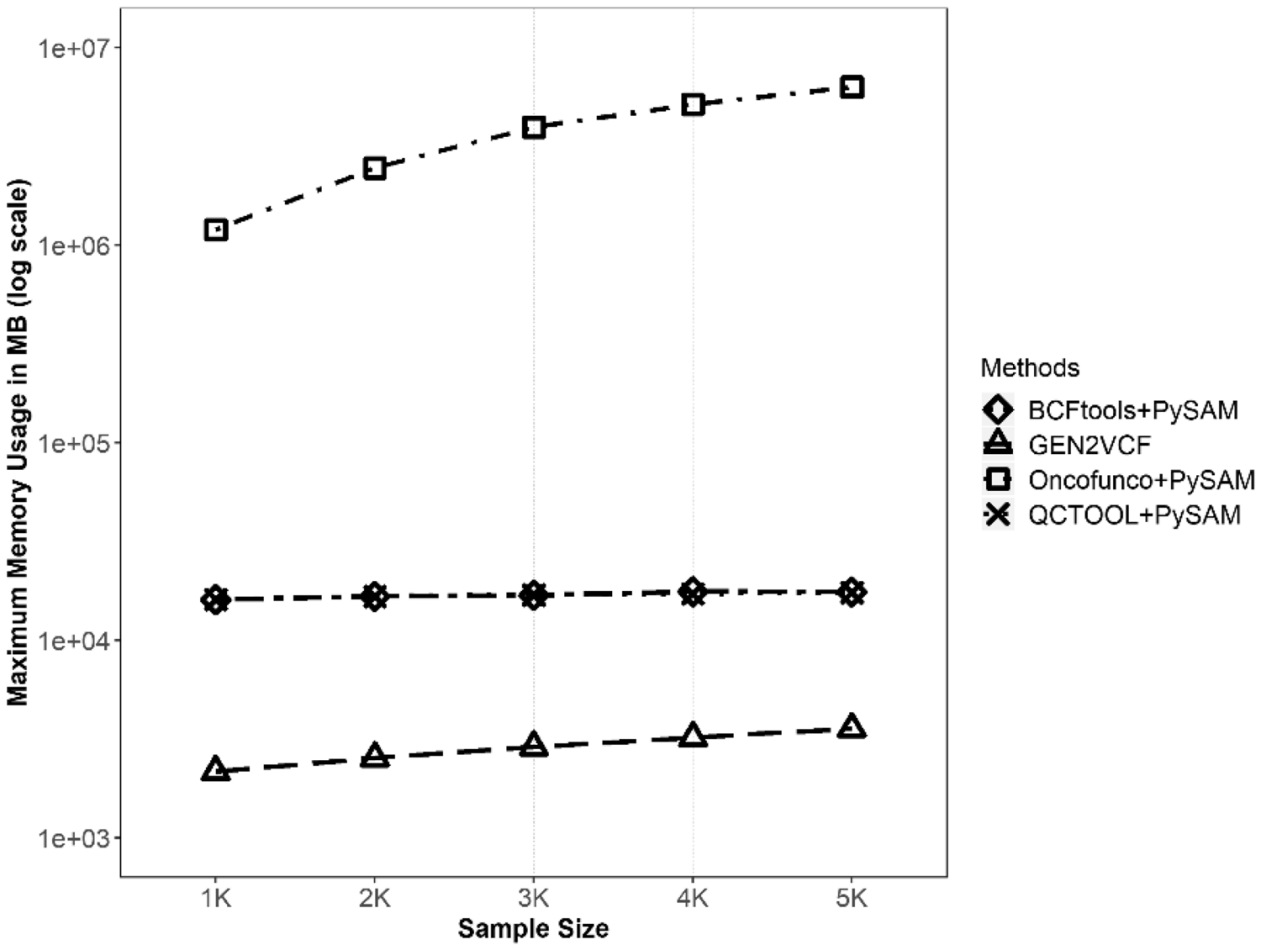
Memory usage comparison among four methods

In terms of memory usage during the conversion process, GEN2VCF had the least memory usage among the methods (Fig. 3). Oncofunco and Pysam use more memory than GEN2VCF to generate the VCF file. When using BCFtools and QCTOOL with PySAM, memory usage was comparable to other methods. For the conversion process, as the sample size increased, the difference in memory usage of other methods increased compared to that of GEN2VCF. When a 1 Mb GEN file with 5000 samples was used as the input, GEN2VCF showed a 7.4–1770-fold decrease in memory usage compared to other methods.

## Discussion

In this study, we developed a new tool to convert the IMPUTE output (GEN format) to VCF with GT, GP, and DS in a single process. The performance of GEN2VCF was compared with three possible pipelines using existing tools. As a result, GEN2VCF showed at least a 1.4-fold decrease in processing time during the conversion. Moreover, GEN2VCF showed the lowest memory usage; at least a 7.4-fold decrease in memory usage was observed when converting a 1 Mb GEN file of 5000 samples. The difference in memory usage was greater by increasing the number of samples for conversion. The memory usage is very important in cases handling millions of samples of whole genome imputed genotypes using a parallel computing environment. Since the maximum memory of a node of a parallel computing environment is limited, large memory usage may produce inefficiencies in the use of computing power for converting GEN files. The increased performance of GEN2VCF was achieved by programming a dedicated conversion software using a high-level C language, minimizing memory usage by processing GEN file line by line appending to a temporary buffer, fast conversion of floating point to string via a custom function. Our results showed that GEN2VCF is an efficient and convenient tool for converting a GEN file to a VCF file with GT, GP, and DS.

In addition to the more efficient performance, GEN2VCF provides users with a convenient option of standard input and output for data processing. This feature is particularly useful in implementing GEN2VCF with various different software packages by piping and redirection. For example, an association test can be performed in a single command line by piping a GEN file management tool (i.e., QCTOOL), GEN2VCF, and association software supporting the VCF. Also, the application can be more efficient in managing storage space if used with a compressed imputation output. Imputed genotype data of millions of samples are typically hundreds of terabytes. For example, the BGEN format can significantly save storage space because it has a smaller file size than files with GEN format (Band and Marchini 2018; Bycroft et al. 2018). Indeed, about half a million samples of whole genome imputation data in the UK Biobank required about 2.1 Tb of file space (Bycroft et al. 2018). In a pipelined command, GEN2VCF can handle a standard output from QCTOOL (which converts BGEN files to GEN files), convert GEN format to VCF with GT, GP, and DS, and then the VCF data can also be redirected to other software packages.

In conclusion, GEN2VCF provides users not only efficient conversion from GEN format to VCF with GT, GP, and DS, but also great flexibility in implementation with other software packages in a pipelined command.

## Data Availability

GEN2VCF: https://bitbucket.org/4shin/division-of-genome-research/src/master.

## References

[CR101] BCFtools: https://samtools.github.io/bcftools/

[CR201] QCTOOL: https://www.well.ox.ac.uk/~gav/qctool/#overview

[CR30] pysam: https://pysam-docs.readthedocs.io/en/latest/

[CR40] Oncofunco: https://github.com/oncogenetics/oncofunco

[CR50] Hail: https://github.com/hail-is/hail

[CR60] Impute v4: https://jmarchini.org/software/

[CR70] SNPTEST: https://mathgen.stats.ox.ac.uk/genetics_software/snptest/snptest.html

[CR80] mach2qtl: https://yunliweb.its.unc.edu/software.html

[CR90] EPACTS: https://genome.sph.umich.edu/wiki/EPACTS

[CR1] Auton A, Brooks LD, Durbin RM, Garrison EP, Kang HM, Korbel JO, Marchini JL, McCarthy S, McVean GA, Abecasis GR (2015). A global reference for human genetic variation. Nature.

[CR2] Band G, Marchini J (2018) BGEN: a binary file format for imputed genotype and haplotype data. bioRxiv

[CR3] Browning Brian L, Browning Sharon R (2016). Genotype imputation with millions of reference samples. Am J Hum Genet.

[CR4] Buniello A, MacArthur JAL, Cerezo M, Harris LW, Hayhurst J, Malangone C, McMahon A, Morales J, Mountjoy E, Sollis E (2019). The NHGRI-EBI GWAS Catalog of published genome-wide association studies, targeted arrays and summary statistics 2019. Nucleic Acids Res.

[CR5] Bycroft C, Freeman C, Petkova D, Band G, Elliott LT, Sharp K, Motyer A, Vukcevic D, Delaneau O, O’Connell J (2018). The UK Biobank resource with deep phenotyping and genomic data. Nature.

[CR6] Das S, Forer L, Schonherr S, Sidore C, Locke AE, Kwong A, Vrieze SI, Chew EY, Levy S, McGue M (2016). Next-generation genotype imputation service and methods. Nat Genet.

[CR7] Feng S, Liu D, Zhan X, Wing MK, Abecasis GR (2014). RAREMETAL: fast and powerful meta-analysis for rare variants. Bioinformatics.

[CR8] Fourment M, Gillings MR (2008). A comparison of common programming languages used in bioinformatics. BMC Bioinform.

[CR9] Ganna A, Genovese G, Howrigan DP, Byrnes A, Kurki M, Zekavat SM, Whelan CW, Kals M, Nivard MG, Bloemendal A (2016). Ultra-rare disruptive and damaging mutations influence educational attainment in the general population. Nat Neurosci.

[CR10] Hoffmann TJ, Witte JS (2015). Strategies for imputing and analyzing rare variants in association studies. Trends Genet.

[CR12] Howie BN, Donnelly P, Marchini J (2009). A flexible and accurate genotype imputation method for the next generation of genome-wide association studies. PLoS Genet.

[CR11] Howie B, Fuchsberger C, Stephens M, Marchini J, Abecasis GR (2012). Fast and accurate genotype imputation in genome-wide association studies through pre-phasing. Nat Genet.

[CR13] Huang L, Li Y, Singleton AB, Hardy JA, Abecasis G, Rosenberg NA, Scheet P (2009). Genotype-imputation accuracy across worldwide human populations. Am J Hum Genet.

[CR14] Khera AV, Chaffin M, Aragam KG, Haas ME, Roselli C, Choi SH, Natarajan P, Lander ES, Lubitz SA, Ellinor PT (2018). Genome-wide polygenic scores for common diseases identify individuals with risk equivalent to monogenic mutations. Nat Genet.

[CR15] Li Y, Willer CJ, Ding J, Scheet P, Abecasis GR (2010). MaCH: using sequence and genotype data to estimate haplotypes and unobserved genotypes. Genet Epidemiol.

[CR16] Liu K, Luedtke A, Tintle N (2013). Optimal methods for using posterior probabilities in association testing. Hum Hered.

[CR17] Loh PR, Palamara PF, Price AL (2016). Fast and accurate long-range phasing in a UK Biobank cohort. Nat Genet.

[CR18] McCarthy S, Das S, Kretzschmar W, Delaneau O, Wood AR, Teumer A, Kang HM, Fuchsberger C, Danecek P, Sharp K (2016). A reference panel of 64,976 haplotypes for genotype imputation. Nat Genet.

[CR19] Moon S, Kim YJ, Han S, Hwang MY, Shin DM, Park MY, Lu Y, Yoon K, Jang HM, Kim YK (2019). The Korea Biobank array: design and identification of coding variants associated with blood biochemical traits. Sci Rep.

[CR20] Visscher PM, Brown MA, McCarthy MI, Yang J (2012). Five years of GWAS discovery. Am J Hum Genet.

